# Reduced Masseter Muscle Area Predicts the 6‐Month Outcome After Mild Traumatic Brain Injury in Older Adults

**DOI:** 10.1002/agm2.70040

**Published:** 2025-08-14

**Authors:** Liang Wu, Yunfei Li, Meng Sun, Nanyu Yao, Zhaofeng Zhang, Weiming Liu

**Affiliations:** ^1^ Department of Neurosurgery Beijing Tiantan Hospital, Capital Medical University Beijing China; ^2^ Department of Nutrition and Food Hygiene, School of Public Health Peking University Beijing China; ^3^ Beijing's Key Laboratory of Food Safety Toxicology Research and Evaluation Beijing China

**Keywords:** aging, computed tomography, masseter muscle, mild traumatic brain injury, sarcopenia

## Abstract

**Objectives:**

Mild traumatic brain injury (mTBI) in older patients is a common condition in neurosurgery, often linked to poor long‐term outcomes, especially when accompanied by frailty. Sarcopenia contributes to this frailty and can be assessed through transverse imaging methods. This study aimed to assess the prognostic value of the masseter muscle cross‐sectional area (MCSA) as determined from admission CT head scans in older patients with mTBI.

**Methods:**

This retrospective study identified older patients with mTBI who were admitted to our hospital from April 2013 to December 2022. The Glasgow Outcome Scale Extended (GOSE) was utilized to assess neurological outcomes at 6 months follow‐up, which were divided into complete recovery (GOSE = 8) and incomplete recovery (GOSE ≤ 7). We measured the average MCSA using admission CT scans and evaluated the consistency of these measurements. Multivariable logistic regression was conducted to evaluate the association between reduced MCSA and 6‐month clinical outcomes in older mTBI patients while adjusting for age, gender, and comorbidity.

**Results:**

The study involved 227 patients, 135 (59.5%) males and 92 (40.5%) females, with a mean age of 74.1 years. 92 (40.5%) had an adverse clinical outcome by the end of follow‐up. The intra‐ and inter‐observer reliability of the MCSA measurements was good to excellent (ICCs = 0.955–0.972 and 0.856–0.892). MCSA decreased with age (Pearson's *r* = −0.290, *p* < 0.001). Males had higher MCSA than females (*p* < 0.001). The optimal MCSA cutoff values for predicting 6‐month clinical outcomes were 358.75 mm^2^ for male and 263.25 mm^2^ for female patients. Reduced MCSA was associated with 6‐month clinical outcomes in univariate and multivariate logistic analyses (OR = 0.131, 95% CI: 0.063–0.273; *p* < 0.001). The MCSA was linearly associated with incomplete recovery (*p* < 0.001, P for nonlinear = 0.127).

**Conclusions:**

MCSA measurements from initial scans were reliable, providing prognostic information that supplemented existing predictors of poor outcomes in older mTBI patients.

## Graphical abstract

MCSA showed an inverse correlation with age upon admission. Male patients demonstrated higher MCSA values compared to female patients. The cutoff points for MCSA based on sex are dividing cutoff values of 358.75 mm^2^ for males and 263.25 mm^2^ for females. MCSA and incomplete recovery have a linear relationship.

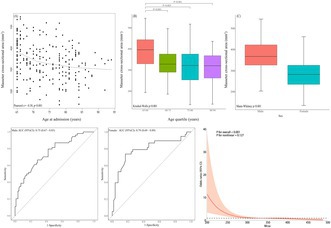

## 1 Introduction

Traumatic brain injury (TBI) has become a major health problem worldwide among adults [1, 2]. The clinical severity of TBI was classified according to the Glasgow Coma Scale (GCS) score, which divided the following categories: severe TBI (GCS score of 3–8), moderate TBI (GCS score of 9–12), and mild TBI (GCS score of 13–15) [3]. The mTBI accounts for up to 90% of all TBI, although this may be an underestimate due to underreporting by patients who do not seek medical attention following the injury [4]. In comparison to other age groups, adults aged 65 years and above are at an elevated risk of sustaining a mTBI [5].

Most recently, mTBI was regarded as leading to symptoms and disability persisting for more than one year, often manifesting as physical, cognitive, and/or emotional symptoms [6]. Various treatments have been developed to improve patient symptoms, including transcranial magnetic stimulation, photobiomodulation, and emerging pharmacological treatments [7, 8]. However, the prognosis for older patients with mTBI remains unfavorable. Various studies have identified multiple prognostic factors in older patients with mTBI [9, 10, 11]. These include clinical factors and individual patient characteristics. Thus, identifying clinically useful prognostic factors and individual patient characteristics is critical to facilitate the best therapeutic approach.

Sarcopenia is defined as a progressive and generalized decline in muscle strength, function, and mass that occurs with increasing age or as a consequence of a disease process [12, 13]. The masseter muscle cross‐sectional area (MCSA), as determined by diagnostic computed tomography (CT) head scans, has been found to correlate with muscle measurements taken at 2 cm below the zygomatic arch in the axial plane. This measurement has been widely studied as a marker of prognosis and mortality in older and trauma patients [14, 15, 16]. Previously, Bosarge et al. found an association between masseter sarcopenia and increased 30‐day mortality in patients with severe TBI [14]. A recent investigative study by Tanabe et al. demonstrated that decreased masseter muscle cross‐sectional area exhibits a significant association with elevated 24‐month post‐traumatic mortality among geriatric patients sustaining traumatic injuries [16].

There is a paucity of studies evaluating the association between reduced MCSA and clinical outcomes in older patients with mTBI. Therefore, the purpose of the present study was to determine sex‐specific cutoff values for MCSA to assess 6‐month outcomes and evaluate the potential impact of MCSA on the functional recovery of patients with mTBI. These findings may help explore new methods for early identification and improvement of clinical outcomes in older patients with mTBI.

## 2 Methods

### 2.1 Study Cohort

**FIGURE 1:**
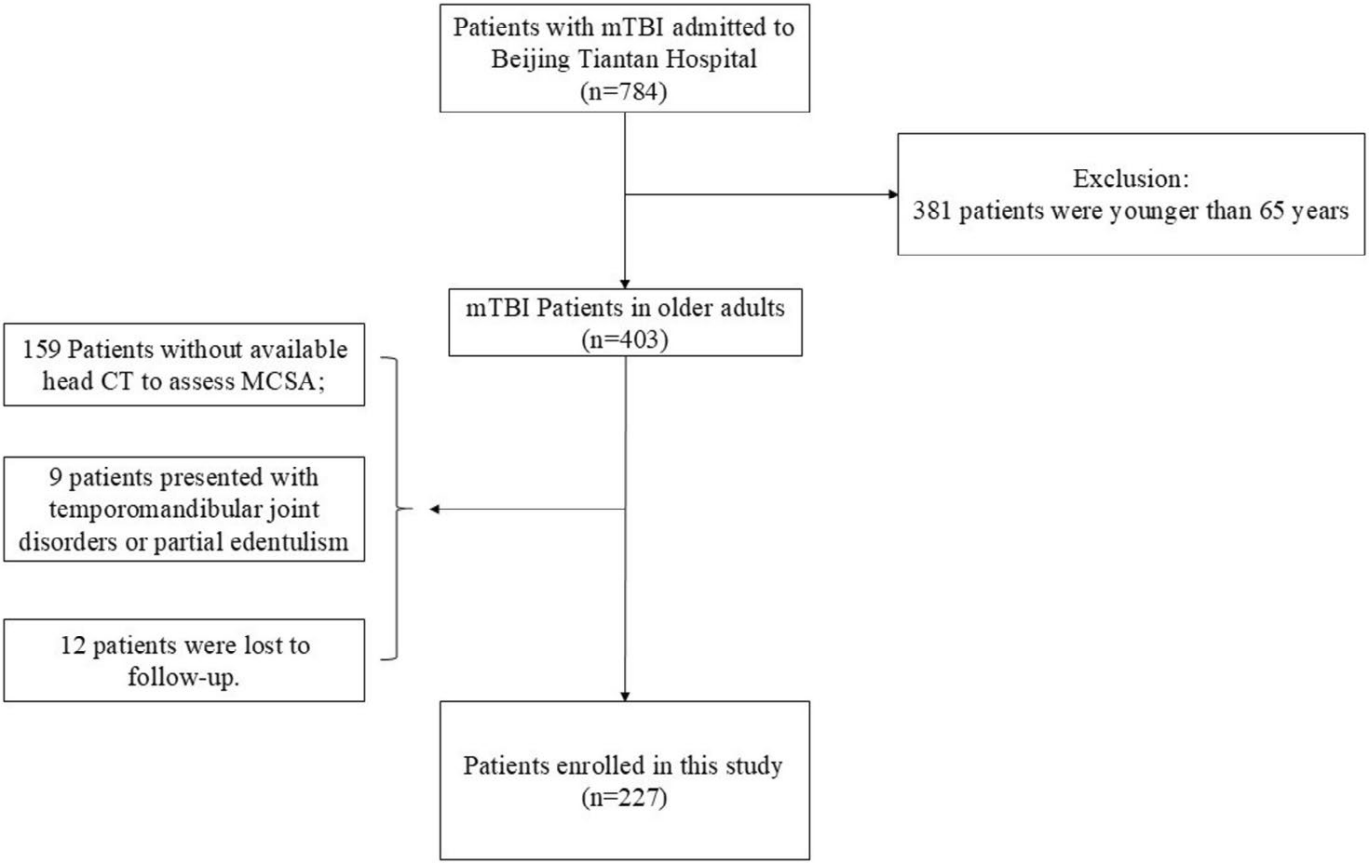
Study flowchart.

This retrospective study included older mTBI patients who presented to Beijing Tiantan Hospital of Capital Medical University between April 2013 and December 2022 (*n* = 227, Figure 1). All included patients met the following criteria: (1) Patients who aged ≥ 65 years; (2) Patients diagnosed with mTBI with a GCS score of 13–15 during hospitalization; (3) Patients who had high‐quality head CT available during hospitalization; and (4) patients with no previously diagnosed neurosurgical disorders. The exclusion criteria were as follows: during hospitalization, (1) CT imaging of the masseter muscles was unavailable; (2) baseline information about the patient could not be accurately obtained; (3) Patients with temporomandibular joint disorders or partial edentulism were excluded; and (4) the CT slice thickness was > 5.0 mm. The study was conducted in accordance with the ethical standards set forth by the Institutional Ethics Review Board of Beijing Tiantan Hospital (approval number: KY2020094‐02). All human participants provided their consent.

### 2.2 Radiological Measurements of Masseter Muscle Cross‐Sectional Area

**FIGURE 2:**
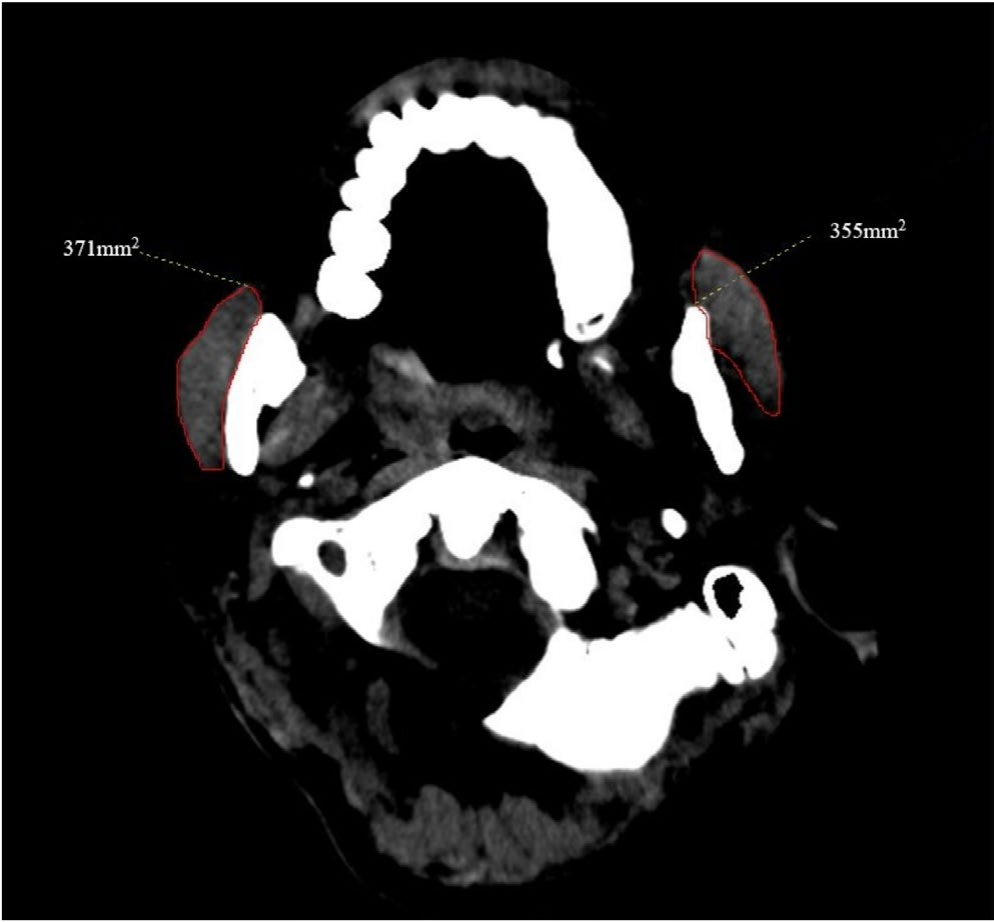
Masseter muscle cross‐sectional area in the axial plane.

The zygomatic arch was identified in each patient in the coronal plane at the level of the base. MCSA was measured at 2 cm below the zygomatic arch in the axial plane [15]. MCSA was recorded as the biggest part of the masseter muscle (Figure 2). The average of the left and right MCSA measurements was used in the analyses. Two neurosurgeons [Yunfei Li. (*n* = 114) and Nanyu Yao. (*n* = 113)] independently measured MCSA. A meeting was convened after collecting measurements from 10 patients to validate the initial agreement among raters. The senior author addressed and resolved any inconsistencies identified during the measurement process (Weiming Liu).

### 2.3 Outcome

The outcome was the Glasgow Outcome Scale Extended (GOSE) score at 6 months postinjury; this score ranged from 1 to 8 and was identified as an effective tool for measuring neurological functional outcomes. A validated questionnaire was used to evaluate the GOSE score. Patients or their caregivers were contacted via telephone at the 6‐month mark to complete the questionnaire. A lower GOSE score was indicative of a poorer recovery outcome. This study used the GOSE score to evaluate the patient outcomes. The clinical outcome was divided into incomplete recovery (GOSE score of 1–7) and complete recovery (GOSE score of 8) [17].

### 2.4 Other Clinical Variables

This analysis involved examining demographic information, clinical data, and injury characteristics, including variables such as age, sex, and Body Mass Index (BMI). Additionally, the age‐adjusted Charlson Comorbidity Index (aCCI) was employed to evaluate the impact of age and comorbidities on disease progression [18]. Past medical history included hypertension, diabetes, coronary atherosclerotic heart disease (CHD), chronic kidney disease, cancer, history of anticoagulant and antiplatelet therapy, and history of smoking and drinking alcohol. The head CT findings included acute subdural hematoma (ASDH), acute epidural hematoma (AEDH), traumatic subarachnoid hemorrhage (TSAH), intracranial hematoma, and skull fracture.

### 2.5 Reliability and Repeatability

To evaluate the consistency of the measurements, the two raters performed a second measurement of MCSA on 30 randomly selected head CT scans from the study's dataset.

### 2.6 Statistical Analyses

Categorical variables were subjected to comparison through the utilization of Pearson's χ^2^ test. Continuous variables were compared using either the Student's t‐test or the Mann–Whitney U test. The data are presented as means with standard deviations or as medians accompanied by interquartile ranges. Differences in variable distributions were analyzed using the Kruskal–Wallis test and Mann–Whitney U test. Pearson's correlation coefficient was applied to evaluate the linear relationship between age and MCSA. The consistency between observers for MCSA measurements was examined using the intraclass correlation coefficient (ICC). The ICCs were reported with the associated 95% confidence intervals (CIs) and interpreted as follows: An ICC value of less than 0.50 was taken to indicate poor reliability; a value between 0.50 and 0.75 was regarded as moderate; a value between 0.75 and 0.90 was good; and a value above 0.90 was deemed to be excellent reliability. Sex‐specific MCSA cutoff values for predicting two‐year mortality were established using ROC curves, with Youden's index optimized to determine the thresholds. Associations between MCSA and patient characteristics were explored through binary logistic regression, with odds ratios (ORs) and CIs used to quantify the relationships. Logistic regression modeling was applied to evaluate the relationship between MCSA and clinical outcomes, with results presented as odds ratios and 95% CIs. Additionally, a restricted cubic spline (RCS) with three knots positioned at the 5th, 50th, and 95th percentiles of MCSA distribution was employed.

All *p*‐values were derived from two‐sided tests and were deemed statistically significant when the associated *p*‐value was less than 0.05. The data were analyzed using R language.

## 3 Results

Two hundred and twenty‐seven patients (135 (59.5%) males and 92 (40.5%) females) were included in this study. The median age of the patients was 74 years. 92 (40.5%) had an adverse clinical outcome by the end of follow‐up. Table 1 reveals the differences in baseline characteristics between the incomplete recovery group and the complete recovery group. A comparison of the incomplete recovery group with the complete recovery group revealed that the former was more likely to be older (76.3 ± 7.5 vs. 72.6 ± 7.0, *p* < 0.001), to have a longer length of stay (13.7 vs. 11.0, *p* = 0.003) and to have a lower MCSA (297.8 ± 80.8 vs. 366.2 ± 77.9, *p* < 0.001). Characteristics stratified by 6 months clinical outcomes are presented in Table 1.

**TABLE 1:** Comparison of clinical factors based on outcome.

Demographic	Total (*n* = 227)	Complete recovery (*n* = 135)	Incomplete recovery (*n* = 92)	*p*
Sex (Male)	135 (59.5%)	81 (60%)	54 (58.7%)	1.000
Age, years	74.1 ± 7.4	72.6 ± 7.0	76.3 ± 7.5	< 0.001
MCSA, mm^2^	338.5 ± 85.8	366.2 ± 77.9	297.8 ± 80.8	< 0.001
Masseter sarcopenia	101 (31.2%)	32 (23.7%)	64 (69.6%)	< 0.001
BMI	23.1 ± 1.7	23.1 ± 1.7	23.2 ± 1.7	0.692
Smoking	65 (28.6%)	41 (30.4%)	24 (26.1%)	0.581
Drinking	38 (17.4%)	27 (20%)	11 (12%)	0.158
GCS	14.7 ± 0.6	14.7 ± 0.5	14.6 ± 0.7	0.033
aCCI	4.34 ± 1.5	4.1 ± 1.4	4.7 ± 1.6	0.008
LOS, (SD), days	12.1 ± 6.4	11.0 ± 5.6	13.7 ± 7.1	0.003
Medical history				
Hypertension	150 (66.1%)	88 (65.2%)	62 (67.4%)	0.840
Diabetes	51 (22.5%)	35 (25.9%)	16 (17.4%)	0.177
CHD	37 (16.3%)	19 (14.1%)	18 (19.6%)	0.359
CKD	8 (3.5%)	1 (0.7%)	7 (7.6%)	0.017
Cancer	15 (6.6%)	8 (5.9%)	7 (7.6%)	0.819
History of anticoagulant	15 (6.6%)	7 (5.2%)	8 (8.7%)	0.439
History of antiplatelet	43 (18.9%)	28 (20.7%)	15 (16.3%)	0.506
Head CT findings				
ASDH	133 (58.6%)	65 (48.1%)	68 (73.9%)	< 0.001
TSAH	115 (50.7%)	61 (45.2%)	54 (58.7%)	0.062
AEH	31 (13.7%)	21 (15.7%)	10 (10.9%)	0.404
Intracranial hematoma	123 (54.2%)	71 (53%)	52 (56.5%)	0.698
Skull fracture	85 (37.4%)	52 (38.8%)	33 (35.9%)	0.758

Abbreviations: aCCI, age‐adjusted Charlson Comorbidity Index; AEH, acute epidural hematoma; ASDH, acute subdural hematoma; BMI, body mass index; CHD, coronary atherosclerotic heart disease; CKD, chronic kidney disease; GCS, Glasgow Coma Scale; LOS, length of stay; MCSA, masseter cross‐sectional area; TSAH, traumatic subarachnoid hemorrhage.

**FIGURE 3:**
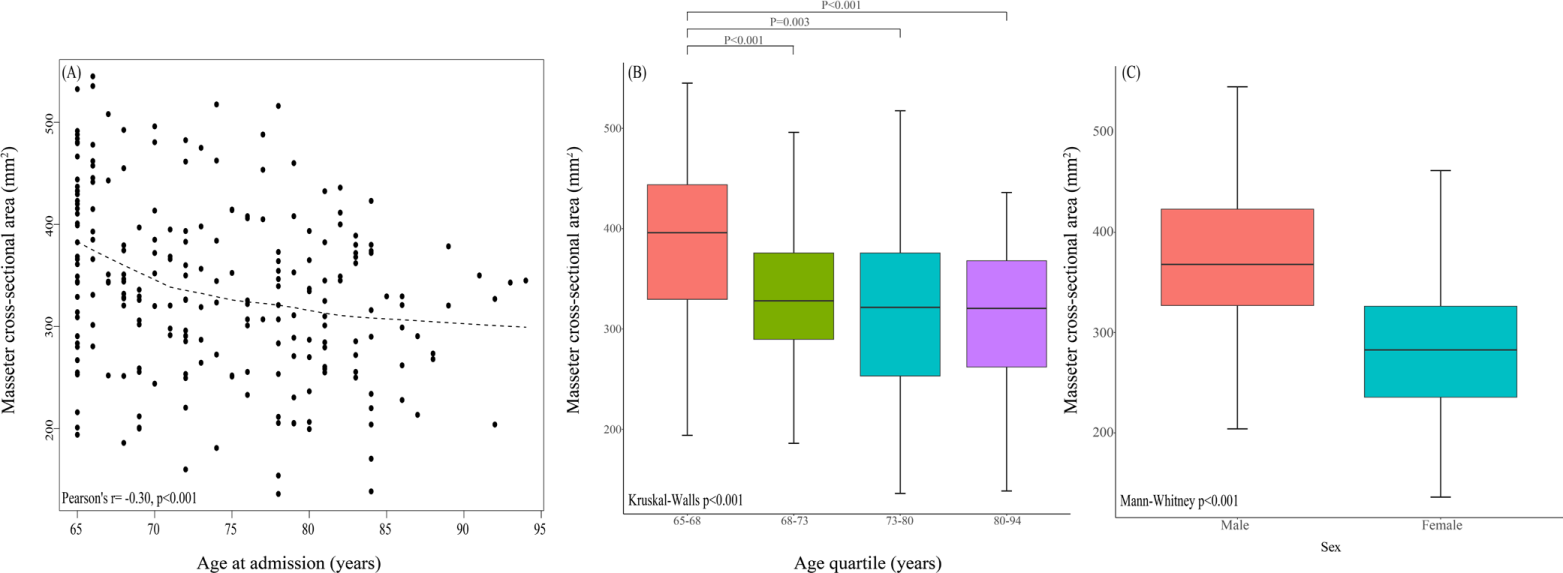
(A) Age‐related distribution of mean masseter muscle cross‐sectional area among 227 patients with mTBI. A locally weighted scatterplot smoothing curve (unbroken line) is shown. Masseter muscle cross‐sectional area was inversely correlated with age at admission. (B) Boxplot of mean masseter muscle cross‐sectional area of each age quartile. Only between‐group comparisons with *p* < 0.05 are shown. (C) Boxplot showing that male patients had higher masseter muscle cross‐sectional area than female patients.

**FIGURE 4:**
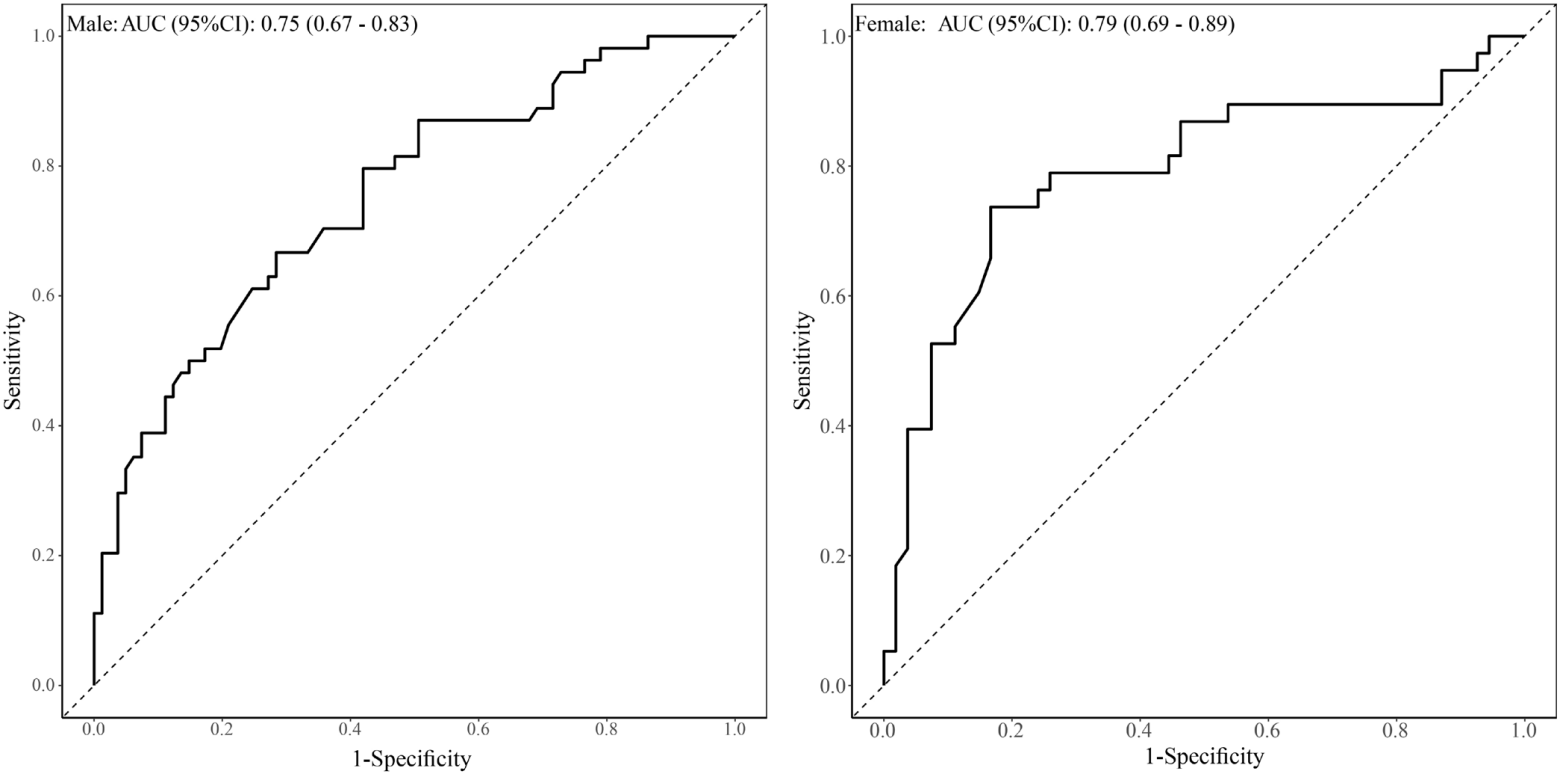
The receiver operating characteristic (ROC) curves for masseter muscle cross‐sectional area (MCSA) were used to predict 6‐month clinical outcomes. The diagonal lines are reference lines demonstrating equal sensitivity and specificity. The resulting MCSA cutoff values were 358.75 mm^2^ for males and 263.25 mm^2^ for females. AUC is the area under the curve.

The reliability between and within observers for MCSA measurements was high to excellent, with intraclass correlation coefficients (ICCs) ranging from 0.955–0.972 for intra‐observer and 0.856–0.892 for inter‐observer assessments (Table S1). Furthermore, MCSA showed an inverse correlation with age upon admission. Male patients demonstrated higher MCSA values compared to female patients (Figure 3). The cutoff points for MCSA based on sex were optimized to predict 6‐month clinical outcomes by maximizing Youden's index (Figure 4), dividing cutoff values of 358.75 mm^2^ for males and 263.25 mm^2^ for females. With these established cutoffs, raters 1 and 2 identified low muscle mass in 32 out of 135 (23.7%) and 64 out of 92 (69.6%) patients, respectively. Patients with MCSA below the sex‐specific thresholds experienced a greater incidence of adverse clinical outcomes compared to those above the thresholds (*p* < 0.001). Patients with MCSA below the sex‐specific cutoff values were also older, had a higher aCCI, and had more CKD. In comparison, factors such as BMI, diabetes status, smoking habits, and alcohol use showed no significant association with MCSA levels above or below the established thresholds (Table 2).

**TABLE 2:** Univariate binary logistic regression analysis of factors associated with Masseter sarcopenia (MCSA below or equal to the sex‐specific cutoff values of 358.75 and 263.25 mm^2^ for male and female patients with mTBI, respectively).

Characteristic	MCSA ≤ cutoff (*n* = 96)	MCSA > cutoff (*n* = 131)	OR (95% CI)
Sex (Male)	37 (38.5%)	55 (42%)	1.154 (0.674 ~ 1.976)
Age, years	75.9 ± 7.2	72.8 ± 7.4	**0.946 (0.912 ~ 0.981)**
BMI	23.1 ± 1.5	23.2 ± 1.9	1.034 (0.887 ~ 1.206)
Smoking	30 (31.2%)	35 (26.7%)	1.247 (0.698 ~ 2.226)
Drinking	16 (16.7%)	22 (16.8%)	0.991 (0.489 ~ 2.007)
aCCI	4.6 ± 1.5	4.2 ± 1.4	0.834 (0.694 ~ 1.003)
Medical history			
Hypertension	62 (64.6%)	88 (67.2%)	1.122 (0.644 ~ 1.955)
Diabetes	19 (19.8%)	32 (24.4%)	1.310 (0.690 ~ 2.487)
CHD	16 (16.7%)	21 (16%)	0.955 (0.469 ~ 1.944)
CKD	7 (7.3%)	1 (0.8%)	**0.031 (0.012 ~ 0.809)**
Cancer	9 (9.4%)	6 (4.6%)	0.464 (0.159 ~ 1.351)
History of anticoagulant	7 (7.3%)	8 (6.1%)	0.827 (0.289 ~ 2.364)
History of antiplatelet	14 (14.6%)	29 (22.1%)	1.665 (0.826 ~ 3.357)

Abbreviations: aCCI, age‐adjusted Charlson Comorbidity Index; BMI, Body Mass Index; CHD, coronary atherosclerotic heart disease; CI, confidence interval; CKD, chronic kidney disease; OR, odd ratio.

**FIGURE 5:**
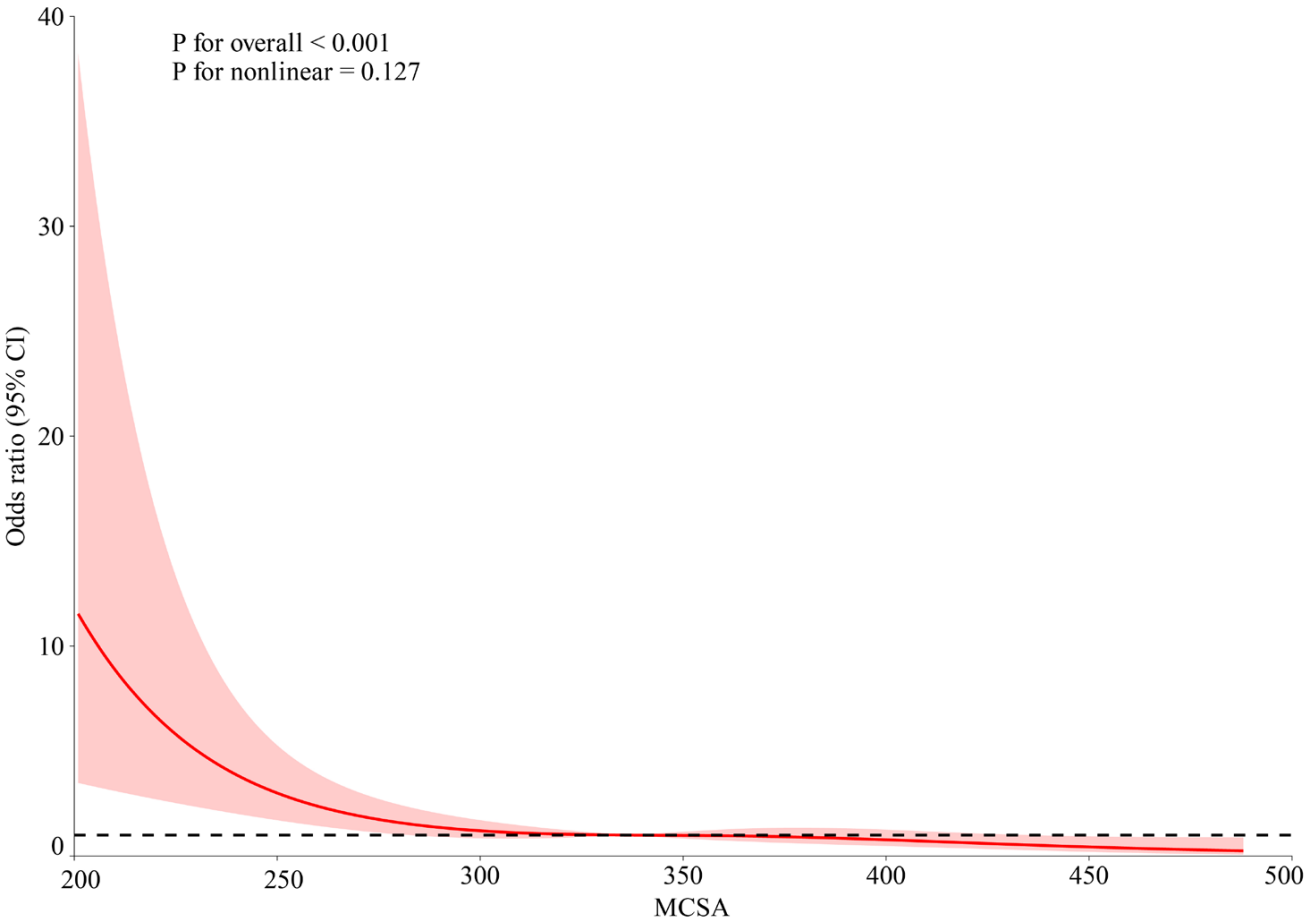
Association of the MCSA and clinical outcome in older patients with mTBI.

After adjusted sex, age, BMI, LOS, hypertension, diabetes, CHD, BMI, Smoking, Drinking, GCS, aCCI, LOS, Hypertension, Diabetes, CHD, CKD, Cancer, History of anticoagulant, History of antiplatelet, ASDH, TSAH, AEH, Intracranial hematoma, Skull fracture, multivariate logistic regression analysis revealed a significantly increased risk of incomplete recovery within 6 months in patients with reduced MCSA (OR = 0.131, 95% CI: 0.063–0.273; *p* < 0.001). Likewise, an increase in MCSA was linked to a reduced risk of incomplete recovery at 6 months (OR = 0.991, 95% CI: 0.988–0.994; *p* < 0.001) as presented in Table 3. In addition, we conducted a multivariable‐adjusted RCS analysis (Figure 5), which illustrates a linear relationship between MCSA and incomplete recovery (*p* < 0.001; nonlinearity *p* = 0.127).

**TABLE 3:** Multivariate logistic regression analysis of the overall outcome of patients with mTBI.

Variables	Model 1		Model 2		Model 3	
	OR (95% CI)	*p*	OR (95% CI)	*p*	OR (95% CI)	*p*
MCSA	0.993 (0.990 ~ 0.995)	< 0.001	0.991 (0.988 ~ 0.994)	< 0.001	0.991 (0.988 ~ 0.994)	< 0.001
MCSA ≤ cutoff	0.136 (0.075 ~ 0.247)	< 0.001	0.150 (0.082 ~ 0.275)	< 0.001	0.131 (0.063 ~ 0.273)	< 0.001

*Note*: Model1: Crude. Model2: Adjust: sex, age. Model3: Adjust: sex, age, BMI, LOS, hypertension, diabetes, CHD, BMI, Smoking, Drinking, GCS, aCCI, LOS, Hypertension, Diabetes, CHD, CKD, Cancer, History of anticoagulant, History of antiplatelet, ASDH, TSAH, AEH, Intracranial hematoma, Skull fracture.

## 4 Discussion

Our findings demonstrated that lower MCSA, observed on admission head CT scans, has a significant association with 6‐month clinical outcomes in older adults with mTBI, providing additional insights beyond established outcome predictors. Patients with reduced MCSA experienced significantly poorer 6‐month clinical outcomes compared to those with MCSA above the defined cutoff (*p* < 0.001). Furthermore, we observed that intra‐ and inter‐rater reliability for MCSA assessments was high, and MCSA values were related to both sex and age.

The psoas muscle cross‐sectional area, identified via abdomen CT scans, remains an important marker for evaluating low muscle mass and is widely recognized as predictive of negative outcomes in oncology and surgical patients [19]. Studies have frequently utilized the axial cross‐sectional area of the psoas muscle. However, due to the rarity of abdominal solid organ injuries after head trauma from falls, routine full‐body CT imaging is rarely recommended [20]. Recent evidence indicates that MCSA measurements correlated with psoas muscle assessment [15]. Prior research by Hu et al. on MCSA in sTBI patients did not cover mTBI, but their findings showed that MCSA values, categorized by one standard deviation relative to sex, correlated with adverse clinical outcomes within 30 days [14].

We sought to evaluate 6‐month clinical outcomes and define sex‐specific cutoff values for diagnosing reduced MCSA in older mTBI patients, shifting focus from short‐term to longer‐term outcomes. Our findings revealed that patients with reduced MCSA (defined as < 368.75 mm^2^ for males and < 263.25 mm^2^ for females) experienced a 2.3‐fold increase in adverse clinical outcome rates relative to those with normal MCSA. Notably, this is the first research to indicate a predictive role of MCSA in older mTBI patients, aligning with prior findings in glioblastoma, head and neck cancers, trauma, and hip fractures, thereby supporting our conclusions [21, 22, 23, 24].

Recently, the association between masseter muscle function and systemic health has attracted research attention. Emerging data suggest that the masseter muscle could act as a reliable marker of a patient's overall physiological reserve. This muscle is mainly composed of type I, IIA, and IIX myosin heavy chains, along with a significant proportion of hybrid fibers containing different myosin types [25]. The content of hybrid fibers is affected by several factors, such as localized stretching and muscle activity. Moreover, this content shows significant variability across different age groups and between sexes [25, 26]. Reduced tension in the masseter muscle is closely linked to lower BMI and reduced hand grip strength [27]. Masseter muscle thickness shows a strong inverse correlation with both occlusive strength and the skeletal muscle index, which are each considered independent risk factors for frailty [28, 29].

As measured through radiological assessment, muscle mass served as a predictor of poor clinical outcomes in older patients with mTBI, independent of their chronological age. This finding suggests that muscle mass is more indicative of biological age, which may account for its predictive value across these age groups. This adds to the growing evidence linking decreased muscle mass to conditions like sarcopenia, frailty, and malnutrition—key geriatric syndromes that are associated with worse patient outcomes [12, 30, 31, 32]. We suggest that understanding the link between low muscle mass and poor clinical outcomes could aid in optimizing resource allocation and developing personalized treatment plans.

Sarcopenia leads to diminished physical capacity and challenges in everyday tasks like sitting and standing. This condition increases the risk of falls and fractures, which can trigger a cascade of complications, including mTBI and venous thrombosis, ultimately resulting in a loss of independent living abilities. Patients with sarcopenia are also prone to malnutrition and reduced immunity, causing endocrine metabolic abnormalities and increasing the risk of influenza, pneumonia, and tumors in older individuals. Previous studies have shown that low muscle mass is a significant cause of falls and injuries, while falls and injuries are also important causes of sarcopenia [33]. Falls and injuries can lead to reduced mobility, fear of falling again, decreased activity, or even hospitalization. These circumstances accelerate the loss of muscle mass in older adults due to reduced activity. On the other hand, mTBI can lead to long‐term symptoms such as dizziness, headaches, insomnia, or even more severe psychological problems, preventing patients from returning to normal life or work. This can accelerate the loss of muscle mass and strength due to reduced physical activity or poor diet, increasing the risk of further brain injury. Therefore, early identification of low muscle mass in older adults with mTBI is essential to improve early care and intervention for this population. This may include progressive resistance training, improved nutritional intake, and protein supplementation to mitigate muscle loss and reduce the risks associated with low muscle mass.

Identifying frailty is crucial in clinical practice, as it serves as a predictor of adverse outcomes and prolonged hospitalization for surgical patients [34, 35]. Our study indicates that head CT scans may serve as a tool for opportunistic screening in older adults with mTBI. Detecting reduced MCSA on these scans at admission should prompt a clinical evaluation to identify the underlying causes of muscle loss, allowing for enhanced geriatric care to improve recovery after treatment and minimize fall risk [36, 37]. Implementing this protocol may allow for more efficient use of resources, potentially enhancing clinical outcomes. However, the effectiveness of these screening methods and targeted therapies in increasing muscle mass has yet to be confirmed. Consequently, identifying targets for the intervention and treatment of mTBI and strategies for prevention has emerged as a crucial area of focus in neurosurgery. Using the MCSA as a predictor to guide prognostic prediction in mTBI patients makes sense for neurosurgeons. Early recognition of low muscle mass is essential for both disease prevention and early intervention. For policy development, it may be possible to popularize or increase rational nutrition for older people and increase outdoor activities to reduce the incidence of sarcopenia, thereby enhancing their mobility, reducing falls, and reducing the incidence of mTBI.

### 4.1 Limitations

This study has inherent limitations due to its retrospective design and potential for selection bias, which may restrict the generalizability of findings. Additionally, the study included only mTBI patients from the Neurotrauma Ward at Beijing Tiantan Hospital, affiliated with Capital Medical University. Although the sample represents the broader demographics of Beijing and nearby regions, reliance on a single institution limits the applicability of the findings. Furthermore, data for patients with non‐brain injuries were unavailable. Several key variables, including admission alcohol level, history of substance use, socioeconomic background, psychiatric history, and postinjury support, were also unavailable, restricting further analysis. Finally, large‐scale, multicenter prospective studies are necessary to confirm the association between lower MCSA and recovery outcomes in older mTBI patients.

## 5 Conclusion

Reduced MCSA was correlated with incomplete recovery in older patients with mTBI. MCSA serves as a straightforward, quick, and dependable measurement tool that provides prognostic insight beyond established predictors of adverse clinical outcomes.

## Author Contributions

**Liang Wu:** conceptualization; **Yunfei Li:** data curation, visualization, writing – original draft; **Nanyu Yao:** data curation; **Meng Sun:** methodology; **Liang Wu, Zhaofeng Zhang, Weiming Liu:** supervision, writing – review and editing.

## Ethics Statement

This study was approved by the Institutional Ethics Review Board of Beijing Tiantan Hospital (approval number: KY2020094‐02). The Clinical trial number is not applicable. All human participants provided their consent.

## Consent

The authors have nothing to report.

## Conflicts of Interest

The authors declare no conflicts of interest.

## Supporting information


**Table S1:** Intra‐ and inter‐observer reliability analysis of the mean MCSA measurements assessed using the ICC. CI, confidence interval; ICC, intraclass correlation coefficient; MCSA, masseter muscle cross‐sectional area. Rater 1.1 = the first rating round of Rater 1, Rater 1.2 = the second rating round of Rater 1, Rater 2.1 = the first rating round of Rater 2, Rater 2.2 = the second rating round of Rater 2.

## Data Availability

The datasets used and/or analyzed during the current study are available from the corresponding author on reasonable request.
