# Supplementary material for: Reduced Masseter Muscle Area Predicts the 6‐Month Outcome After Mild Traumatic Brain Injury in Older Adults
Source: Aging Med (Milton). 2025 Aug 14;8(4):294–302. doi: 10.1002/agm2.70040 (PMC12399341; doi:10.1002/agm2.70040)
Supplement: Supplementary file 1 — Table S1: Intra‐ and inter‐observer reliability analysis of the mean MCSA measurements assessed using the ICC. CI, confidence interval; ICC, intraclass correlation coefficient; MCSA, masseter muscle cross‐sectional area. Rater 1.1 = the first rating round of Rater 1, Rater 1.2 = the second rating round of Rater 1, Rater 2.1 = the first rating round of Rater 2, Rater 2.2 = the second rating round of Rater 2. [file AGM2-8--s001.docx]

**Supplementary Table 1.** Intra- and inter-observer reliability analysis of the mean MCSA measurements assessed using the ICC

| Comparison | ICC (95% CI) |
| --- | --- |
| **Intra-observer** |  |
| Rater 1.1 v. Rater 1.2 | 0.955 (0.931-0.971) |
| Rater 2.1 v. Rater 2.2 | 0.972 (0.957-0.982) |
| **Inter-observer** |  |
| Rater 1.1 v. Rater 2.1 | 0.892 (0.832-0.93) |
| Rater 1.2 v. Rater 2.2 | 0.856 (0.776-0.908) |
| Rater 1.1 v. Rater 2.2 | 0.878 (0.809-0.922) |
| Rater 1.2 v. Rater 2.1 | 0.863 (0.786-0.912) |

MCSA = masseter muscle cross-sectional area, ICC = intraclass correlation coefficient, CI = confidence interval. Rater 1.1 = the first rating round of Rater 1, Rater 1.2 = the second rating round of Rater 1, Rater 2.1 = the first rating round of Rater 2, Rater 2.2 = the second rating round of Rater 2.
